# Generative Models for Active Vision

**DOI:** 10.3389/fnbot.2021.651432

**Published:** 2021-04-13

**Authors:** Thomas Parr, Noor Sajid, Lancelot Da Costa, M. Berk Mirza, Karl J. Friston

**Affiliations:** ^1^Wellcome Centre for Human Neuroimaging, Queen Square Institute of Neurology, London, United Kingdom; ^2^Department of Mathematics, Imperial College London, London, United Kingdom; ^3^Department of Neuroimaging, Centre for Neuroimaging Sciences, Institute of Psychiatry, Psychology & Neuroscience, King's College London, London, United Kingdom

**Keywords:** active vision, generative model, inference, Bayesian, oculomotion, attention

## Abstract

The active visual system comprises the visual cortices, cerebral attention networks, and oculomotor system. While fascinating in its own right, it is also an important model for sensorimotor networks in general. A prominent approach to studying this system is active inference—which assumes the brain makes use of an internal (generative) model to predict proprioceptive and visual input. This approach treats action as ensuring sensations conform to predictions (i.e., by moving the eyes) and posits that visual percepts are the consequence of updating predictions to conform to sensations. Under active inference, the challenge is to identify the form of the generative model that makes these predictions—and thus directs behavior. In this paper, we provide an overview of the generative models that the brain must employ to engage in active vision. This means specifying the processes that explain retinal cell activity and proprioceptive information from oculomotor muscle fibers. In addition to the mechanics of the eyes and retina, these processes include our choices about where to move our eyes. These decisions rest upon beliefs about salient locations, or the potential for information gain and belief-updating. A key theme of this paper is the relationship between “looking” and “seeing” under the brain's implicit generative model of the visual world.

## Introduction

This paper reviews visual perception, but in the opposite direction to most accounts. Normally, accounts of vision start from photons hitting the retina and follow a sequence of neurons from photoreceptor to visual cortex (and beyond) (Goodale and Milner, 1992; Wallis and Rolls, 1997; Riesenhuber and Poggio, 1999; Serre et al., 2007; DiCarlo et al., 2012). At each step, we are told about the successive transformation of these data to detect edges, contours, objects, and so on, starting from a 2-dimensional retinal image and ending with a representation of the outside world (Marr, 1982/2010; Perrett and Oram, 1993; Carandini et al., 2005). In this paper, we reverse this account and ask what we would need to know to generate a retinal image. Our aim is to formalize the inference problem the brain must solve to explain visual data. By framing perceptual inference or synthesis in terms of a forward or generative model, we arrive at the space of hypothetical explanations the brain could call upon to account for what is happening on the retina (Helmholtz, 1878 (1971); MacKay, 1956; Neisser, 1967; Gregory, 1968, 1980; Yuille and Kersten, 2006).

The motivation for this perspective comes from formalisations of brain function in terms of (active) inference (Friston et al., 2017; Da Costa et al., 2020). The idea is that the brain makes use of an implicit model of how sensory data are generated. Perception is then the inversion of this model to find the causes of our sensations (Von Helmholtz, 1867; Gregory, 1980; Doya, 2007). Here, the term ‘inversion’ refers to the use of (approximate) Bayesian inference to compute posterior probabilities that represent (Bayesian) beliefs about the world. This is an inversion in the sense that we start with a model of how the world generates sensations and ask what the sensations we obtain tell us about the world. Central to this is the bidirectionality inherent in inference. It is this bidirectionality that manifests in neurobiology (Friston et al., 2017a; Parr and Friston, 2018b), where messages are passed reciprocally between neural populations

In a sense, everything we have said so far only brings us to the point that vision is not just ‘bottom-up’ but that it has an important “top-down” element to it—which is uncontroversial (Zeki and Shipp, 1988; Lee and Mumford, 2003; Spratling, 2017). However, we take this one step further and argue that if the messages passed up visual hierarchies are the inversion of a (top-down) generative model, then all we need to do is understand this model, and the ascending pathways should emerge naturally, under some neuronally plausible message passing scheme. For this reason, we will focus upon the problem that the visual brain must solve and will not concern ourselves with the details of its solution, reserving this for a future paper.

Perceptual inference is just one part of the story (Ferro et al., 2010; Andreopoulos and Tsotsos, 2013; Zimmermann and Lappe, 2016; Pezzulo et al., 2017). We only sample a small portion of our sensory environment at any one time. In the context of vision, this depends upon where our retina is pointing. This tells us that, to generate a retinal image, we need to take account of how we choose where to look (Ognibene and Baldassarre, 2014). The problem of deciding where to look, and of influencing the biophysical processes required to implement these decisions, are also inference problems. The first relies upon the notion of planning as inference (Botvinick and Toussaint, 2012). Here, we treat alternative action sequences as a set of hypotheses. To select among these, we must weigh prior beliefs about the best course of action against the evidence sensory data afford to each plan. Under active inference, the priors are assumed to favor those plans for which there is a high expected information gain (Lindley, 1956; Itti and Koch, 2000; Itti and Baldi, 2006; Friston et al., 2015; Yang et al., 2016). In short, we have to plan our visual palpation of the world in a way that allows us to construct a scene in our heads that best predicts “what would happen if I looked over there” (Hassabis and Maguire, 2007; Schmidhuber, 2010; Zeidman et al., 2015; Mirza et al., 2016).

The process of implementing these plans is also an inference problem but cast in a slightly different way. In its variational form, approximate Bayesian inference can be framed as optimisation. The inference is deemed optimal when a lower bound on the Bayesian model evidence—the probability of data given a model—is maximized (Beal, 2003; Winn and Bishop, 2005; Dauwels, 2007). While this lower bound can be maximized by closing the gap between the bound and the evidence, it can also be maximized by selecting data that cohere with the model, increasing the evidence itself (c.f., self-evidencing (Hohwy, 2016)). The implication is that we can use action to change the data generating process to fit the world to the model, in addition to fitting the model to the world. For active vision (Wurtz et al., 2011), this means predicting the proprioceptive data we might expect from the oculomotor muscles if a given eye movement is made. Maximizing the evidence then means changing—through contraction or relaxation—muscle lengths until the predicted input is achieved. This can be regarded as a formalization of the equilibrium point hypothesis for motor control (Feldman and Levin, 2009), which posits that all we need do is specify some desired setpoint that can be fulfilled through brainstem (or spinal) reflexes.

To address these issues, we divide this paper into two main sections. First, we deal with the ‘seeing’ problem. Here, we start from a given environment (e.g., a room we might find ourselves in) and our location in it and ask what pattern of retinal cell activity we would predict. This depends upon the contents of that environment (e.g., the furniture in the room) and the location and geometry of those contents. In addition, it depends upon where we are in the environment, which way we are facing, and the orientation of our eyes relative to our head. We then turn to the ‘looking’ problem, and its constituents: where to look and how to look there. By formulating looking and seeing as generative models, we reduce the problems to a series of conditional dependencies. As our interest here is in active vision as implemented by the brain, we keep in mind the anatomical manifestations of these conditional dependencies as connections between neural populations.

## Seeing

**Figure 1:**
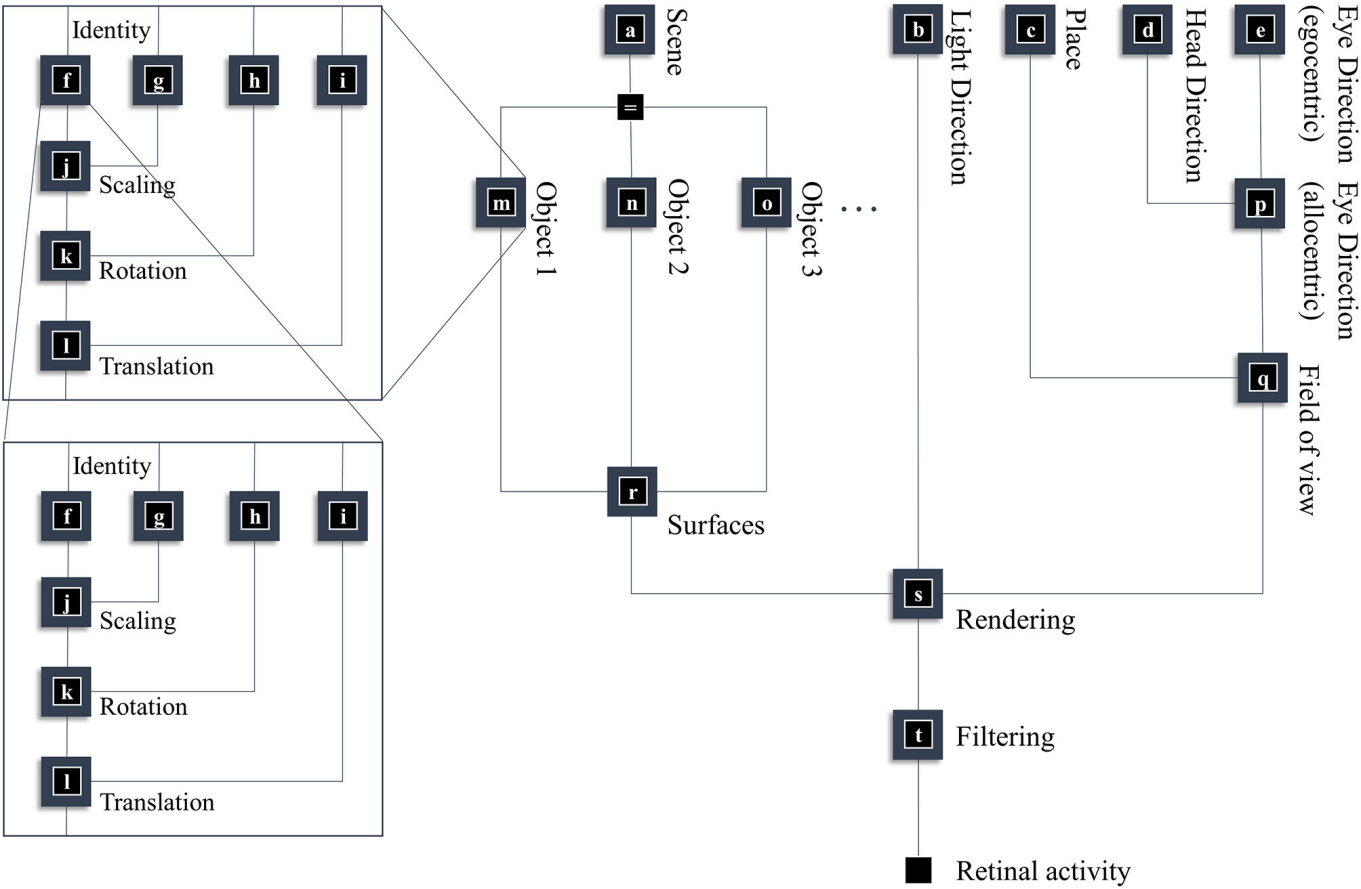
A generative model for seeing. This figure offers a summary of a model that generates a retinal image. Starting from the scene in which we find ourselves, we can predict the objects we expect to encounter. These objects may be recursively defined, through identifying their constituent parts and performing a series of geometric (affine) transformations that result in the configuration of parts, the scaling and rotation of this overall configuration, and the placement of these objects in an allocentric reference frame. The nested parts of the graphical structure (on left hand side) indicate this recursive aspect, e.g., an object is defined by an identity, and scaling, rotation and translation parameters; furthermore, it may be composed of various sub-objects each of which has these attributes. To render a retinal image, we also need to know where the retina is and which way it is pointing (i.e., the line of sight available to it). This depends upon where we are in the environment, our heading direction, and where we choose to look. Subsequent figures unpack the parts of this model in greater detail.

In this section, our aim is to generate a retinal image. Figure 1 provides an overview of the generative model in (Forney) factor graph format (Loeliger, 2004; Loeliger et al., 2007; Forney and Vontobel, 2011; Laar and Vries, 2016; de Vries and Friston, 2017; van de Laar and de Vries, 2019). As we will appeal to this formalism throughout, we will briefly describe the conventions. As the name suggests, this graphical notation depends upon factorizing the problem into a series of smaller problems. If we assume a set of latent (or hidden) variables *x* that generate our retinal image *y*, we can write down a probability distribution that can be decomposed according to the conditional dependencies in the generative model. For example:

(1) $$\begin{array}{l} P\;\left(y,x^{1},x^{2},x^{3},\ldots \right)=P\;\left(y|x^{1}\right)\;P\;\left(x^{1}|x^{2},x^{3}\right)\;P\;\left(x^{2}|x^{4}\right)\ldots \end{array}$$

To construct a factor graph of Equation (1), we would take each factor on the right-hand side and draw a square. We then draw a line coming out of this square for every variable that appears inside the factor. If that variable appears in another factor, we connect the line to the square representing the other factor. For those used to looking at Bayesian networks—where edges denote factors—it is worth emphasizing that edges in a factor graph denote random variables. This may seem a little abstract. However, we will go through the components of the factor graph in Figure 1 in detail over the next few sections. The important thing to begin with is that the upper left of the factor graph relates to scene and object identity. In contrast, the upper right deals with locations and directions. The separation of these explanatory variables offers our first point of connection with neuroanatomy, as this closely resembles the “what” (ventral) and “where” (dorsal) visual streams that support object and spatial vision, respectively (Mishkin et al., 1983). The sections on The Ventral Stream and The (Extended) Dorsal Stream deal with these pathways, and the section on The Retinocortical Pathway deals with their convergence.

### The Ventral Stream

This section focuses upon the identity and shape of the things causing our visual sensations. From a neurobiological perspective, the structures involved in object and scene identification are distributed between the occipital and temporal lobes (Kravitz et al., 2013). The occipitotemporal visual areas are referred to as the ‘what’ pathway or the ventral visual stream. The occipital portion of the pathway includes cells with receptive fields responsive to concentric circles (Hubel and Wiesel, 1959) and gratings (Hegdé and Van Essen, 2007). The temporal portion contains cells with more abstract response properties, relying upon more specific feature configurations that are invariant to size, view, or location (Deco and Rolls, 2004; DiCarlo et al., 2012). We will start from the more abstract (temporal) end of this pathway and work our way toward the simpler features at the occipital end.

**Figure 2:**
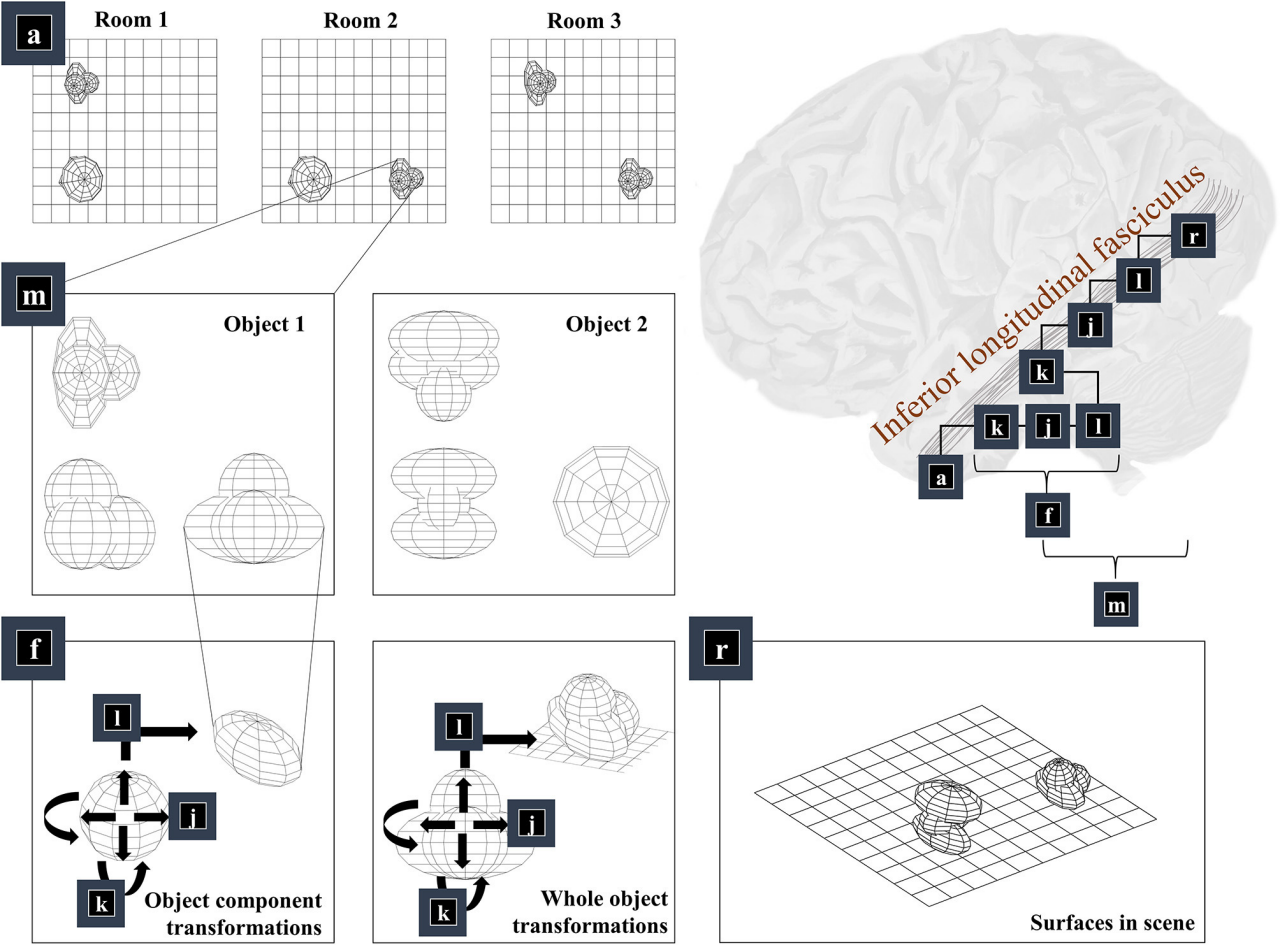
The “what” pathway. This figure focuses upon part of the factor graph in Figure 1. In the upper right, this factor graph is reproduced as it might be implemented neuroanatomically. Here, the factors are arranged along the occipitotemporal “what” pathway, which loosely follows those cortical areas superficial to a white matter tract called the inferior longitudinal fasciculus. The panels on the left show the sequence of steps implemented by these factors. Factor **a** is the distribution over alternative rooms (or scenes) we could find ourselves in. This implies a categorical distribution assigning a probability to each of the rooms provided in the example graphics. Conditioned upon being in a room, we may be able to predict which objects are present. Two example objects are shown (from three orthogonal views). The conditional probability distribution for the geometry of the objects given the room is given by the **m** factor, which is here broken down into several constituent factors. First, we need to know the identity of the objects in the room (**f**), which we operationalise in terms of the configuration of the parts of that object. Specifically, we decompose **f** into a series of transformations (**k**-**l**) applied to a set of spheres, which represent the constituents of the object. In principle, we could have used other objects in place of spheres, or could have applied this procedure recursively, such that the constituents of an object can themselves be decomposed into their constituents. Once we have our object, we can apply the same transformations (**k-l**) to the whole to position it our room. We do this for each object in the room, eventually coming to a representation of all the surfaces in the scene (**r**), shown in the lower right panel.

The first thing we need to know, to generate an image, is the environment in which we find ourselves. A schematic of a simple environment is shown in Figure 2, which shows three possible rooms—each of which contains two objects that can appear in different locations. If we knew which of these rooms we were in, we could predict which objects were present. This is approximately the same structure as used in previous accounts of scene construction in a 2-dimensional world (Mirza et al., 2016). It has neurobiological validity as evidenced by the proximity of the inferotemporal cortex, associated with object recognition (Logothetis and Sheinberg, 1996; Tanaka, 1996), to the parahippocampal gyrus, associated with recognition of places (Epstein et al., 1999), hinting at how the brain might represent dependencies between scenes and their constituent objects.

Once we know which objects we expect to be present, we can associate them with their 3-dimensional geometry. To generate these objects, we assume they are constructed from simpler structures—for the purposes of illustration, spheres. Figure 1 illustrates the recursive aspect to this, where the object factor (**m**) is decomposed into a series of geometric (affine) transformations applied to a structure as identified by the object identity factor (**f**), which itself can be decomposed into a series of transformations of simpler features. In other words, an object's geometry depends upon the configuration of its features (e.g., the legs and surface of a table), but these features can themselves depend upon configurations of simpler features (Biederman, 1987). Implicit in this perspective is that the scene itself is simply the highest level of the recursion, comprising features (objects) that themselves comprise simpler features. Figure 2 illustrates this idea graphically.

Taking a step back, we need to be able to represent the shape of a feature before we can start applying transformations to it. One way of doing this is to construct a mesh. Meshes specify the vertices of the surfaces that comprise an object (Baumgart, 1975), effectively setting out where we would expect to find surfaces. This is the form shown in the graphics of Figure 2—where we have omitted occluded surfaces for visual clarity. Note that we have taken a subtle but important step here. We have moved from discussing categorical variables like scene or object identity and have started working in a continuous domain. At this point, we can apply geometric transforms to our objects. The first is the scaling of an object (factor **j**), which is a simple linear transform using a matrix (*S*) whose diagonal elements are positive scaling coefficients along each dimension. This is applied to each coordinate vector of our mesh. Expressing this as a factor of a probability distribution, we have:

(2) $$\begin{array}{l} P\;\left(x^{j}|x^{f},x^{g}\right)=\delta \;\left(S(x^{g})x^{f}-x^{j}\right)\\ \;S\;\left(\left[\alpha ,\beta ,\gamma \right]\right)=\left[\begin{array}{ccc} e^{\alpha } & & \\ & e^{\beta } & \\ & & e^{\gamma } \end{array}\right] \end{array}$$

The *x* variables represent the edges in the graph of Figure 1. The superscripts indicate the factor from which the edge originates (i.e., the square node above the edge). The *x*^*f*^ variable includes the coordinates of the vertices of each surface of the object. This is transformed based upon the scaling in each dimension (in the *x*^*g*^ variable) to give the scaled coordinates *x*^*j*^. The scaling variables are treated as log scale parameters. This means we can specify factor **g** to be a Gaussian distribution without fear of negative scaling. However, we could relax this constraint and allow for negative scaling (i.e., reflection). In addition, we could include off-diagonal elements to account for shear transforms. In Equation (2), δ is the Dirac delta function—a limiting case of the (zero-centered) normal distribution when variance tends to zero. It ensures there is non-zero probability density only when its argument is zero. This is a way of expressing an equality as a probability density. We could have used a normal distribution here, but for very large objects, with many surfaces, the associated covariance matrices could become unwieldy. It is simpler to absorb the uncertainty into the priors over the (log) scaling parameters.

Our next step is to apply rotations to the object. Here, we use a rotation matrix (*R*) that has the form:

(3) $$\begin{array}{l} P\left(x^{k}|x^{j},x^{h}\right)=\delta \left(R(x^{h})x^{j}-x^{k}\right)\\ \;R([\theta ,\phi ,\varphi ])=\left[\begin{array}{ccc} \cos(\phi )\cos(\varphi ) & -\cos(\phi )\cos(\varphi ) & \sin\left(\phi \right)\\ \cos(\theta )\sin(\varphi )+\sin\left(\theta \right)\sin\left(\phi \right)\cos\left(\varphi \right) & \cos(\theta )\sin(\varphi )-\sin\left(\theta \right)\sin\left(\phi \right)\sin\left(\varphi \right) & -\sin\left(\theta \right)\cos\left(\phi \right)\\ \sin(\theta )\sin(\varphi )-\cos(\theta )\sin\left(\phi \right)\cos\left(\varphi \right) & \sin(\theta )\cos(\varphi )+\cos(\theta )\sin\left(\phi \right)\sin\left(\varphi \right) & \cos\left(\theta \right)\cos\left(\phi \right) \end{array}\right] \end{array}$$

As in Equation (2), we use the Dirac delta distribution such that the rotated coordinates can only plausibly be the original coordinates, rotated. This defines the **k** factor.

Finally, we translate the objects (factor **l**). This is simply a matter of adding the same vector to all vertices of the mesh and centring a Dirac delta distribution for *x*^*l*^ on this value. Figure 2 shows two applications of these three operations that give us the components of object 1 (lower left panel) and that place object 1 in a particular place in our scene (lower middle panel). The factor **r** simply concatenates the surfaces from all objects such that *x*^*r*^ is simply a list of surfaces.

Is there any validity to the idea that the brain might generate objects with a series of geometrical transforms of this sort? Evidence in favor of this comes from two lines of research. One is in psychological experiments which show that, during object recognition, reaction times scale with the angle of rotation that would have to be performed to bring that object into a familiar configuration (Cooper and Shepard, 1973; Tarr and Pinker, 1989)—suggesting a form of implicit mental rotation. This is consistent with the idea that the brain optimizes its model through updating beliefs about the degree of rotation until it best fits the data at hand.

The second line of evidence is from neurophysiological studies into invariance of neural responses to different properties. To understand the relevance of invariant representations, note that the transforms we have described do not commute with one another. To see this, consider what would happen if we were to rotate the sphere before rescaling it. The implication is that, if there are objects whose identity is preserved with changes in its geometry, we should expect to see different sorts of invariance emerge at different stages along the visual hierarchy. At the highest levels, we might expect neural responses to be consistent for an object, no matter how it is oriented, scaled, or translated. As we descend toward the occipital lobe, we might anticipate these invariances being lost, in sequence. This is exactly what happens (Rust and DiCarlo, 2010; Grill-Spector and Weiner, 2014; Tacchetti et al., 2018), with inferotemporal cortical cells responding to specific objects, regardless of their size, position (Ito et al., 1995), or the angle from which they are viewed (Ratan Murty and Arun, 2015). As we move toward the occipital cortex, neurons become more sensitive to the rotation of an object (Gauthier et al., 2002; Andresen et al., 2009). On reaching areas V2-V4 of the early visual cortex, the receptive fields of neurons are many times smaller than those in inferotemporal cortex (Kravitz et al., 2013). This means they respond only when a stimulus is in a specific region of space, implying loss of translation invariance. Evidence that the brain inverts a model of this sort comes from studies illustrating that the activity of (feedforward) convolutional neural networks trained on visual data—which implicitly account for the requisite transforms—aligns with gamma-band activity in visual cortices (Kuzovkin et al., 2018). This frequency band is crucial in ascending neural message passing (Bastos et al., 2015) associated with model inversion (Friston, 2019).

While we chose affine transforms for simplicity, it is worth emphasizing that the generative model is highly non-linear. This is most striking for the recursive part of the ventral stream model, which alternates between linear operations (affine transformations of the shapes) and non-linear operations (selection between shapes). To invert this kind of model, one would employ a linear operation to undo the affine transformations for each component of an object. On finding the log likelihood of the inverted shape for each component, one could compute a posterior by adding the log prior for each component and taking a non-linear softmax transform. This is then repeated for the next level of the recursion, eventually returning a categorical distribution over plausible objects that could be causing visual data. The alternation between linear and non-linear operations—in the inversion of this model—could explain why deep learning architectures, that alternate in this way, have been so successful in machine vision. Non-affine transformations could be incorporated through using a spatial basis set to deform the objects or their components—analogous to the models employed for spatial normalization in image analysis (Arad et al., 1994; Ashburner and Friston, 1999; Shusharina and Sharp, 2012). This would involve adding additional factors into the ventral stream model that represent these deformations but would not change the overall anatomy of the model.

In summary, we have gone from prior beliefs about the room we occupy to beliefs about the objects in that room. These are decomposed into their constituent parts, and the surfaces that define these parts. At the occipital end of the pathway, we have a set of surfaces. Taken individually, these surfaces could belong to any object. Each occupies a smaller portion of space than the complete objects. This means that, in the process of generating the geometric structures we will need for vision, we have traversed the ventral visual pathway from the large, abstract receptive fields of the inferior temporal cortices to the smaller, simpler receptive fields of the occipital lobe.

A final consideration for this section is the consequence of damage to the brain structures implementing this generative model. Ventral visual stream lesions give rise to an interesting category of neuropsychological syndromes, broadly referred to as agnosia (Adler, 1944; Benson and Greenberg, 1969; Greene, 2005). There are many variants of agnosia, but common to all is a failure to recognize something. Visual agnosia is an inability to recognize objects, sometimes restricted to specific categories. For example, prosopagnosia is a form of visual agnosia specific to faces (Sacks, 2014). Generative modeling offers a useful perspective on agnosia, as any lesions to the ventral stream impair the capacity of a model to predict the visual data that would be anticipated if a given object were present. If we assumed that a given lesion removed all neurons involved in representing object 1 from Figure 2 or cut the connections that predicted the surfaces anticipated when object 1 is present, we could generate as many images as we wanted by sampling from the generative model without ever generating one characteristic of object 1. Without this hypothesis available to the brain, it is unable to invert the data-generating process to arrive at the conclusion that object 1 is present. Despite this, it might still be possible to identify its constituent parts, particularly if these parts are like those found in other objects.

### The (Extended) Dorsal Stream

**Figure 3:**
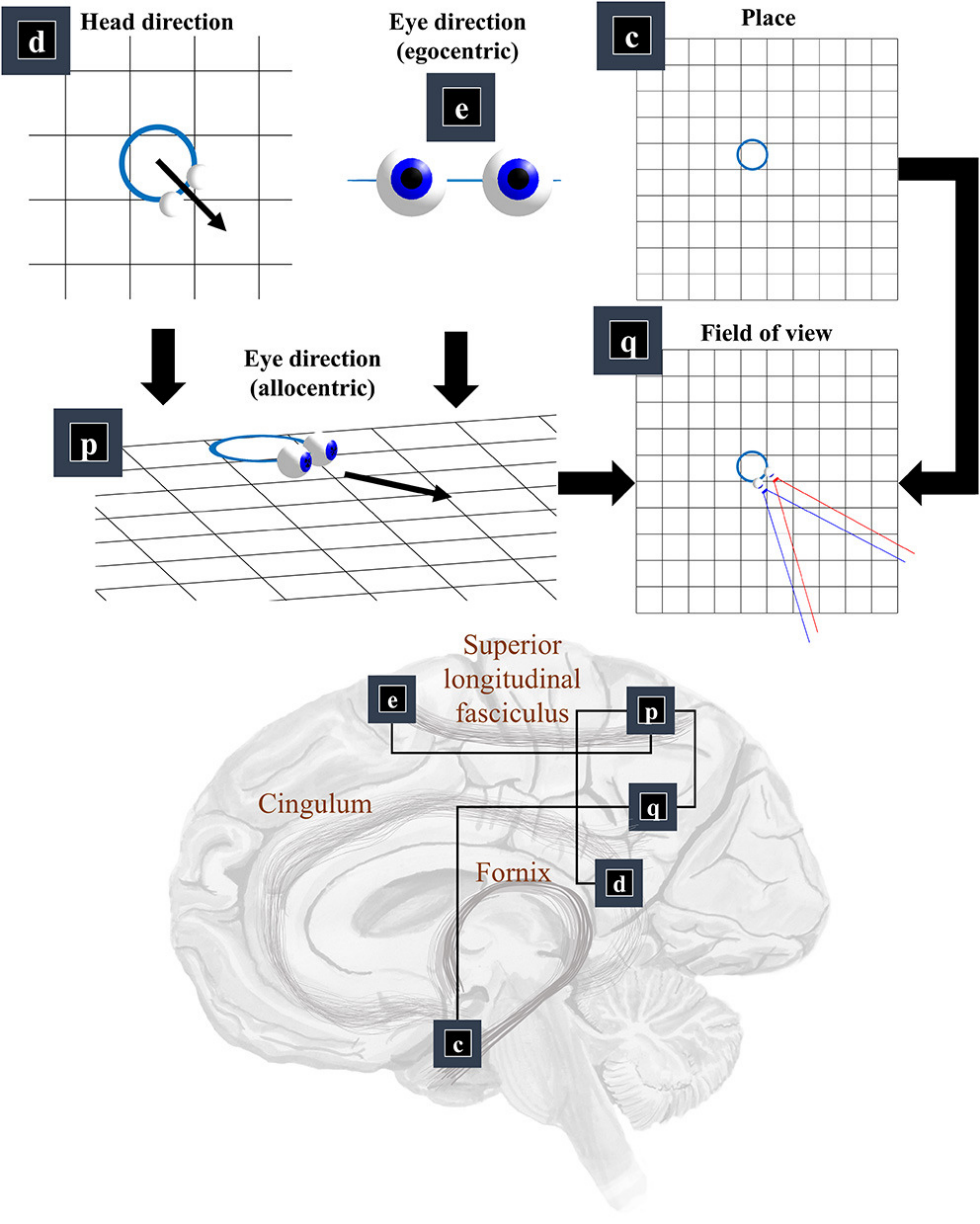
The “where” pathway. This figure shows the factors that conspire to generate a field of view. This shows how the allocentric head and egocentric eye-directions (factors **d** and **e**) can be combined to compute an allocentric eye-direction (factor **p**). When this vector is placed so that it originates from the place (factor **c**) we find ourselves in, we have our field of view (factor **q**). The graphic in the lower part of this figure maps the associated factors onto the brain structures thought to be involved in representing these variables. The frontal eye-fields (factor **e**) and the retrosplenial cortex (factor **d**), both project to the parietal cortex (factor **p**), which includes regions sensitive to allocentric eye-directions. This communicates with temporoparietal regions (factor **q**), which are also accessible to hippocampal outputs (factor **c**) via a pathway comprising the fornix, mammillothalamic, and cingular white matter tracts.

Now that we know the positions and orientations of the surfaces in our scene, we need to know the same for our retina. To know where our retina is, the first thing we need to know is where our head is in allocentric space. In other words, where we are in our environment. The part of the brain most associated with this is outside of the classical visual brain. It is the hippocampal formation that famously contains place (and grid) cells, which increase their firing rate when an animal is in specific places (or at repeating intervals) in an environment (Moser et al., 2008). Figure 3 illustrates this by placing factor **c—**prior beliefs about place—in the medial temporal lobe.

We need more than the location of the head to be able to locate the retina. First, we need to know which way the head is facing. Head-direction cells, which fire maximally when an animal is oriented along a given direction in its environment, are found distributed throughout the brain (Taube et al., 1990; Taube, 1995; Blair et al., 1998). Specifically, they are found in the constituents of the Papez circuit (Papez, 1995), originally thought to mediate emotional responses. Together, the place and head-direction tell us where the eyes are, but they do not pinpoint the retinal location. For this, we also need to know the direction in which the eyes are pointing. Combining the head-direction (factor **d**) with the egocentric eye-direction (factor **e**), we can compute the allocentric eye-direction (factor **p**). With information about place, this gives us our field of view (factor **q**).

Expressed as a probability distribution, factor **p** is:

(3) $$\begin{array}{l} P\;\left(x^{p}|x^{d},x^{e}\right)=\delta \;\left(x^{d}+x^{e}-x^{p}\right) \end{array}$$

This ensures the allocentric eye-direction is given by the angle of the head plus the angle of the eyes relative to the head. We can augment this for each eye, to allow for their convergence—i.e., that the directions of the left and right eye are not parallel to one another. Factor **q** is a little more complicated but involves constructing arrays representing locations of retinal cells or, more simply, locations in front of the lens that, if light were to pass through the location and reach the lens, would refract to a given retinal photoreceptor (or group of photoreceptors). We generate one array for each eye. We make a simplification here in that we assume we are dealing with a small foveal area such that we can ignore the global topography of the retina. As such, we treat the array of cells as uniformly spaced. A more complete retinal model would take account of the log-polar organization (Javier Traver and Bernardino, 2010), in which the density of photoreceptors decreases with retinal eccentricity—i.e., distance from the fovea. This array, along with the location of the lens, gives us our field of view. Taking the outermost cells from each array, we simply project from the lens, through that location. This generates the blue and red lines in the **q** panel of Figure 3. The *x*^*q*^ variables are tuples, for each element of the retinal array, containing the location and a unit vector representing its preferred angle of incidence.

The classical ‘where’ pathway involves the occipitoparietal cortices. Figure 3 shows how the factors needed to compute a field of view could converge upon the parietal lobe, assuming we assign factor **q** to the temporoparietal cortices. Interestingly, these regions have been associated with the ability to take another point of view in several different senses. Electrical stimulation of these regions on the right side of the brain can induce out of body experiences (Blanke et al., 2002), where people feel as if they are observing the world from a vantage point outside of their body. We also talk informally about seeing things from another person's point of view. This relates to theory of mind, and the ability to infer another's perspective at a more abstract level. These functions are also associated with the temporoparietal cortices (Abu-Akel and Shamay-Tsoory, 2011; Santiesteban et al., 2012). The implication is that the same machinery may be involved in taking a viewpoint, both in the literal and metaphorical sense, and that this machinery is housed in the temporoparietal region. Some have argued that this representation of viewpoint is central to the first-person perspective that underwrites conscious experience (Seth, 2009; Williford et al., 2018).

The retrosplenial cortex is a good candidate for factor **d**, given its role in relating visual ‘where’ data with head-direction (Marchette et al., 2014; Shine et al., 2016). Specifically, it is responsive to where we have to look to find stable, unambiguous, landmarks (Auger et al., 2012). Lesions to this region impair the representation of head-direction in other parts of the brain—notably the anterior thalamus—even in the presence of clear visual landmarks (Clark et al., 2010). Neuropsychological evidence supports this assignment, as lesions to the retrosplenial cortex can cause a form of topographical disorientation, where patients lose their sense of direction (Aguirre and D'Esposito, 1999).

The translation from head-centered eye-direction to a world-centered reference frame (i.e., factors **e** and **p**) is consistent with the connections from the frontal eye fields to the parietal lobe. These connections are underwritten by a white matter tract known as the superior longitudinal fasciculus (Makris et al., 2005; Thiebaut de Schotten et al., 2011). The parts of the brain connected by this tract are referred to as the attention networks (Corbetta and Shulman, 2002; Szczepanski et al., 2013)—identified through their recruitment in attentional tasks during neuroimaging studies. The frontal eye fields (Bruce et al., 1985) and intraparietal sulcus (Pertzov et al., 2011) both contain neurons sensitive to eye position, in different coordinate systems.

In summary, the generation of a line of sight depends upon the head location and direction, and the position of the eyes relative to the head. These are represented in the medial temporal lobe, the frontal lobe, and medial parietal structures. The convergence of axonal projections from these regions to the lateral parietal lobe provides the dorsal visual stream with key information, which can be reciprocally exchanged with the occipital cortices. While we have adopted the rhetoric of “what” and “where” streams, it is interesting to note that the controllable aspects of the generative model all relate to the “where” stream. This provides a useful point of connection to a complementary framing of the two visual streams. Under this alternative perspective (Goodale and Milner, 1992), the ventral stream is thought to support perception, while the primary role of the dorsal stream is to inform action. This view is informed by neuropsychological findings (Goodale et al., 1991), including the ability of those with dorsal stream lesions to see objects they cannot grasp, and the ability of those with lesions to other parts of the visual cortices grasp objects they could not see.

### The Retinocortical Pathway

So far, we have generated a set of surfaces, and a field of view. The final challenge of our ‘seeing’ generative model is to convert these to a pair of retinal images. This is analogous to the process of rendering in computer graphics (Shum and Kang, 2000). There are many ways to implement sophisticated rendering schemes, and a review of these is outside the scope of this paper. We will outline one way in which a simple form of rendering may be implemented and consider whether this has neurobiological correlates.

For any given retinal photoreceptor, we can trace an imaginary line out through the lens of the eye and ask which surface it will first encounter. If it does not pass through any surface, this means there is nothing that can reflect light in the direction of that cell, and the receptor will not be activated. However, if it does encounter a surface, we must determine the intensity of light that surface reflects in the direction opposite to our imaginary line. This is similar to the ray tracing method in computer graphics (Whitted, 1980), and depends upon the rendering equation (Kajiya, 1986):

(4) $$\begin{array}{l} P\;\left(x^{s}|x^{r},x^{q},x^{b}\right)=\delta \;\left(\Lambda \left(x^{q},x^{r},x^{b}\right)-x^{s}\right)\\ \;\Lambda \left(u,v,z\right)=\eta \;\left(u,v\right)\times \\ \text{> }\left(\underset{\text{Ambient}}{\underset{︸}{\alpha \;\left(u,v\right)}}+\int_{S}\underset{\text{Reflected}}{\underset{︸}{\Lambda \left(v,w,z\right)\;\beta \;\left(u,v,w\right)dw}}\right) \end{array}$$

The variables in the conditioning set are the light direction (*x*^*b*^), as a unit vector, and tuples containing information about the surfaces of objects (*x*^*r*^) and the retinal cells (*x*^*q*^). The η function acts as an indicator as to whether a line passing through the lens, that would refract light to a specific retinal cell (*u*), intersects with a point on a surface (*v*) before reaching any other surface. It is one if so, and zero otherwise. The α function plays the role of ambient lighting, and we assume this is a constant for all surfaces, for simplicity. The β function determines the proportion of light reaching a surface from other sources (*w*)—e.g., reflected off other surfaces (*S*)—that is reflected toward *u*. The recursive structure of the integral part of this expression resembles the recursive marginalization that underwrites belief-propagation schemes (Frey and MacKay, 1998; Yedidia et al., 2005). Recursive expressions of this sort can usually be solved either analytically—e.g., through re-expression in terms of an underlying differential equation—or numerically. In principle, we could construct a factor graph like that of Figure 1, using the β functions as our factors, determining the dependencies between the level of illumination of each surface. The integral includes all surfaces *S* that could reflect light to surface *v*. To simplify, we ignore the dependencies between surfaces, and assume a single level of recursion (i.e., surfaces reflect light to the retina, but the light incident on a surface originates directly from the light source). This means we choose *S* = *z*, so that Equation (4) simplifies to:

(5) $$\begin{array}{l} \Lambda \left(u,v,z\right)=\eta \;(u,v)\;(\alpha \left(u,v\right)+\eta \;(v,z)\;\alpha \;\left(v,z\right)\;\beta \;\left(u,v,z\right)) \end{array}$$

The key differences between different approaches to generating images rest upon the choice of β. We follow the approach outlined in (Blinn, 1977):

(6) $$\begin{array}{l} \beta \;\left(u,v,z\right)=\underset{\text{Diffuse}}{\underset{︸}{c_{1}\max\left(0,v_{n}\cdot z\right)}}+\underset{\text{Specular}}{\underset{︸}{c_{2}{\left(v_{n}\cdot \frac{u_{n}+z}{\sqrt{\left(u_{n}+z\right)\cdot \left(u_{n}+z\right)}}\right)}^{c_{3}}}} \end{array}$$

**Figure 4:**
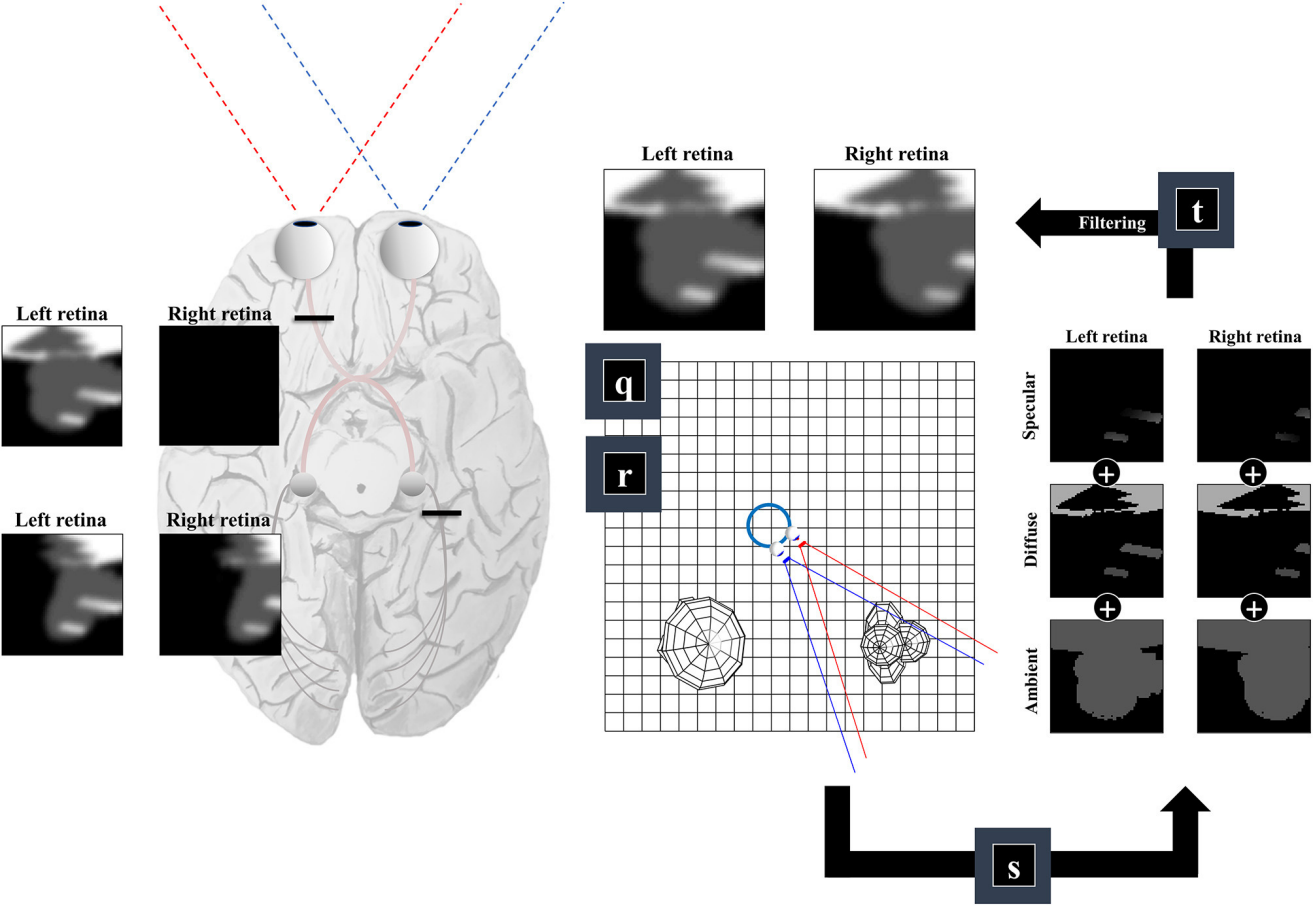
The retinocortical pathway. This figure takes the results from Figures 2, 3 and combines these to arrive at images in each retina. From factors **r** and **q** we have our field of view and the surfaces it captures. We can then project from each retinal cell (shown as pixels in the retinal images) to see whether any surface is encountered. For the first surface we reach, we combine the ambient, diffuse, and specular lighting components (factor **s**). This depends upon factor **b** from Figure 1, which provides a lighting direction. Once we have the sum of these lighting components, we apply a blurring (factor **t**) to the image to compensate for the artificial high frequency components introduced by our simplifications. Practically, this is implemented by finding the coefficients of a 2-dimensional discrete cosine transform, multiplying this by a Gaussian function centered on the low frequency coefficients, and then performing the inverse transform. Note that the final image is inverted across the horizontal and vertical planes. This is due to the light reflecting off surfaces on the temporal visual fields being propagated to the retina on the nasal side, and vice versa (with the same inversion in the superior and inferior axis). The fact that the same surface can cause activation of both the right and left retina implies a divergence in the predictions made by parts of the brain dealing in surfaces (e.g., striate cortices) about retinal input. The graphic on the left illustrates the two sorts of visual field defect resulting from this divergence—either interrupting the influence of any surface on one retina (upper image) or interrupting the influence of a subset (e.g., the right half) of all surfaces on either retina (lower image).

Equation (6) uses the subscript *n* to indicate (normalized) unit vectors drawn from the *u* and *v* tuples (which also include the coordinates of the origins of these vectors). For *u*_*n*_, this vector is parallel to the line from the lens outwards—in the opposite direction to the light that would be refracted to a specific group of cells on the retina. For *v*_*n*_ it is the normal unit vector to the surface in question^1^. Equation (6) includes a diffuse term, which allows for light to be reflected equally in all directions, where the amount reflected depends upon the angle of incidence. In Figure 4, we see how this lighting component catches some surfaces but not others, and the way in which it induces shadows (via multiplication with the η function). The specular component accounts for the relationship between the angle of incidence and the angle of reflectance from a surface (Phong, 1975). To gain some intuition for this term, imagine shining a torch into a mirror. The reflection will appear maximally bright when the angle between the torch and the normal to the mirror is equal to the angle between your eye and the normal to the mirror and will rapidly decay on moving either eye or torch.

A simplification made in the above is to treat the lens as a point, neglecting the fact that there are a range of angles of light that could be focused upon a given cell in the retina. In reality, neighboring photoreceptors may encounter photons reflected from the same point on a surface. To account for the artificial high frequency components introduced during this discretisation of space, we apply a blurring effect (factor **t**) This is based upon a discrete cosine transform followed by attenuation of those coefficients corresponding to these high frequencies followed by the inverse transform. Specifically, we multiply the coefficients by a Gaussian function centered on the low frequency components. An interesting consequence of this relates to the inversion of this model. Undoing this process would mean replacing the high frequency components. This enhancement might give the appearance of edge detection—a common role afforded to cells in the early visual pathway with center-surround receptive fields (Crick et al., 1980; Marr et al., 1980). In addition, it could account for the sensitivity of early visual neurons to specific spatial frequencies, and the widespread use of grating stimuli and Gabor patches in experiments designed to interrogate these cells (Mahon and De Valois, 2001).

An important feature of this generative model is the fact that surfaces on the left of the head (in egocentric space) are projected to the right side of both retinas. Similarly, surfaces on the right of the head are projected to the left side of both retinas. This is interesting in the sense that there are two sorts of deficit we could induce. As shown on the left of Figure 4, we could disconnect one retina, precluding surfaces from either side of space from generating an image on this side. This generates images consistent with monocular blindness. Alternatively, by precluding any surface on one side of space from causing retinal cell activation, we lose activity on the same side of both retinas—i.e., a homonymous hemianopia. This maps to the deficits found on lesions to the retinocortical pathway before and after the optic chiasm, respectively (Lueck, 2010; Wong and Plant, 2015). This highlights the inevitability of these visual field defects following lesions to the visual pathway, under the assumption that the brain uses a model that represents the same surfaces as causes of data on both retinas.

The generative model ultimately must generate the data it seeks to explain. For our purposes, these data are the signals sent from the retina to the visual cortex. However, it is possible to take this further and to specify the kinds of generative model used within the retina itself. Attempts to do this have focused upon a prior belief about the smoothness of input across the retina and have provided useful accounts of efficient retinal processing as predictive coding (Srinivasan et al., 1982; Hosoya et al., 2005).

## Looking

**Figure 5:**
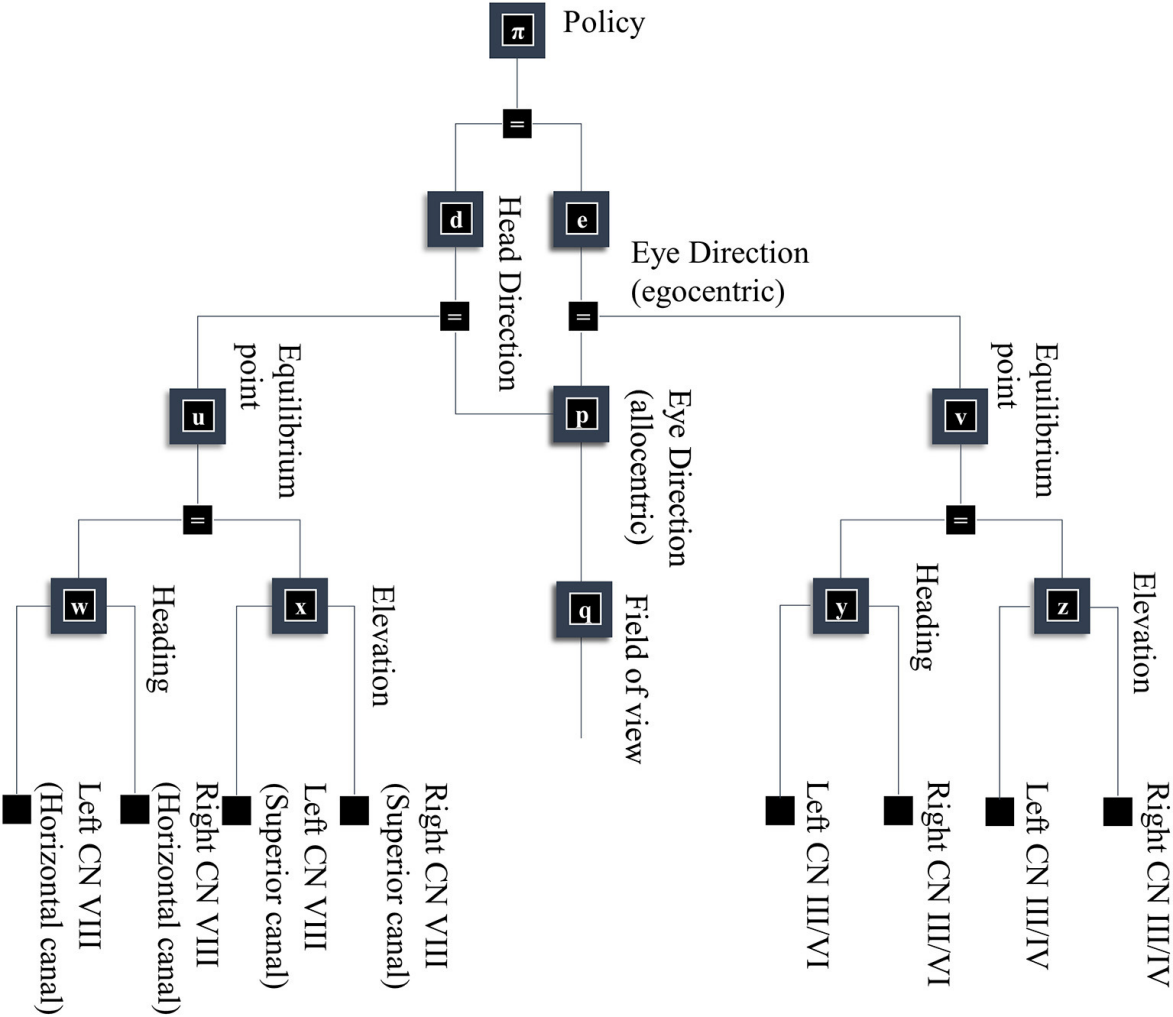
A generative model for looking. This figure builds upon part of the model shown in Figure 1. Specifically, it unpacks some of the other sources of data resulting from the eye and head-direction factors and includes a policy variable that determines priors over these variables. These depend upon dynamical systems. This means predicting an equilibrium point (or attractor) that the eyes or head are drawn toward. These dynamics may be divided into changes in the elevation or heading angles. For head movements, the velocity of the head causes changes in the semi-circular canals in the inner ear, communicated to the brain by cranial nerve (CN) VIII. For eye movements, the position and velocity of the eyes give rise to proprioceptive signals due to stretch of the oculomotor muscle tendons, communicated to the brain by CN III, IV, and VI.

As alluded to above, retinal data depends not just upon what is “out there” in our environment, but upon where we direct our gaze. Figure 5 takes factors **d** and **e** from Figure 1, and conditions these upon a policy variable. This accounts for the fact that our choices determine where our eyes and our head are facing. In addition, Figure 5 shows some of the non-visual sensory modalities that result from these explanatory variables. These depend upon dynamical systems, as the motion of the head and eyes cause changes in vestibular and proprioceptive modalities. This is of particular importance when thinking about movement as the solution to an inference problem. When acting so as to minimize any discrepancy between predicted and realized sensations, thereby maximizing the evidence for a model, the predicted consequences of action become central to the performance of that action. The section on The Brainstem unpacks the generation of proprioceptive data from the oculomotor muscles and the relationship to the oculomotor brainstem. The section on The Basal Ganglia then focuses upon formulation of prior beliefs about the policy—and its neurobiological manifestation in the oculomotor loops of the basal ganglia. Together, these can be seen in the spirit of agenda-driven perspectives (Ballard and Zhang, 2020) on action, where we unpack a selected policy into the set of processes that must be initiated at lower levels of a model to execute or realize that policy.

### The Brainstem

This section focuses upon the biophysics of oculomotion that underwrites implementations of saccadic eye movements. Modeling the eyes is relatively straightforward. They tend to move together^2^ and can be described using Newton's second law applied to rotational forces (McSpadden, 1998). This describes the relationship between a torque τ applied at radius *r* to a point mass *m* and an angle θ:

(7) $$\begin{array}{l} \;\tau =mr^{2}\ddot{\theta }\\ \Rightarrow \int_{0}^{\infty }\tau \left(r\right)dr=\ddot{\theta }\int_{0}^{\infty }m\left(r\right)r^{2}dr \end{array}$$

The second line of this equation relates the first to a solid object, where the torque and the density (*m*(*r*)) of the object can vary with the radius. The oculomotor muscles that generate torques insert into the surface of the eyeballs, meaning we can simplify Equation (7) as follows:

$$\begin{array}{l} \tau \left(r\right)=\tau \delta \left(r-r_{\max}\right)\Rightarrow \\ \;\tau =J\ddot{\theta } \end{array}$$

(8) $$\begin{array}{l} J≜\int_{0}^{\infty }m\left(r\right)r^{2}dr \end{array}$$

The term *J* in the final line is a constant known as the “moment of inertia.” Equation (8) implies the following equations of motion:

(9) $$\begin{array}{l} \begin{array}{l} \theta ≜\left[\begin{array}{c} \theta \\ \overset{∙}{\theta } \end{array}\right]\\ \overset{∙}{\theta }=f\left(\phi ,\theta \right)≜\left[\begin{array}{c} \overset{∙}{\theta }\\ J^{-1}\tau \left(\phi \right) \end{array}\right] \end{array} \end{array}$$

All that is left is to provide a functional form for the torque. We can choose this such that the eyes come to rest at an angle ϕ:

(10) $$\begin{array}{l} \tau \left(\phi ,\theta ,\overset{∙}{\theta }\right)=\phi -\theta -\kappa \overset{∙}{\theta } \end{array}$$

This is analogous to the torque associated with a swinging pendulum. The constant κ determines the damping, which precludes large oscillations around ϕ. We can interpret ϕ as a target or setpoint, in the spirit of the equilibrium point hypothesis of motor control (Feldman and Levin, 2009). Now that we have the equations of motion of the eye—noting that we have a single equation for both eyes to enforce conjugacy^3^. (Parr and Friston, 2018a)—we must detail the sensory consequences of these movements. These are given as follows:

(11) $$\begin{array}{l} g\left(\theta ,\omega \right)≜\left[\begin{array}{l} \theta -\frac{1}{2}\omega \\ \overset{∙}{\theta }\\ \theta +\frac{1}{2}\omega \\ \overset{∙}{\theta } \end{array}\right] \end{array}$$

Here, ω represents the convergence of the eyes, accommodating the fact that the angle between the two can vary. The first two rows relate to the left eye, and the last two to the right. Equation (11) assumes a direct mapping from the angular positions and velocities of each eye to the proprioceptive input from the oculomotor muscles, consistent with the role of II and Ia sensory afferents (Cooper and Daniel, 1949; Cooper et al., 1951; Ruskell, 1989; Lukas et al., 1994), respectively.

Converting Equations (9–11) to factors of a probability distribution, we have:

(12) $$\begin{array}{l} \begin{array}{c} P\;\left({ẋ}^{v}|x^{v},x^{e}\right)=N\;\left(f\left(x^{v},x^{e}\right),\Pi_{f}\right)\\ P\;\left(y^{y},y^{z}|x^{v}\right)=N\;\left(g\left(x^{v},\omega \right),\Pi_{g}\right) \end{array} \end{array}$$

**Figure 6:**
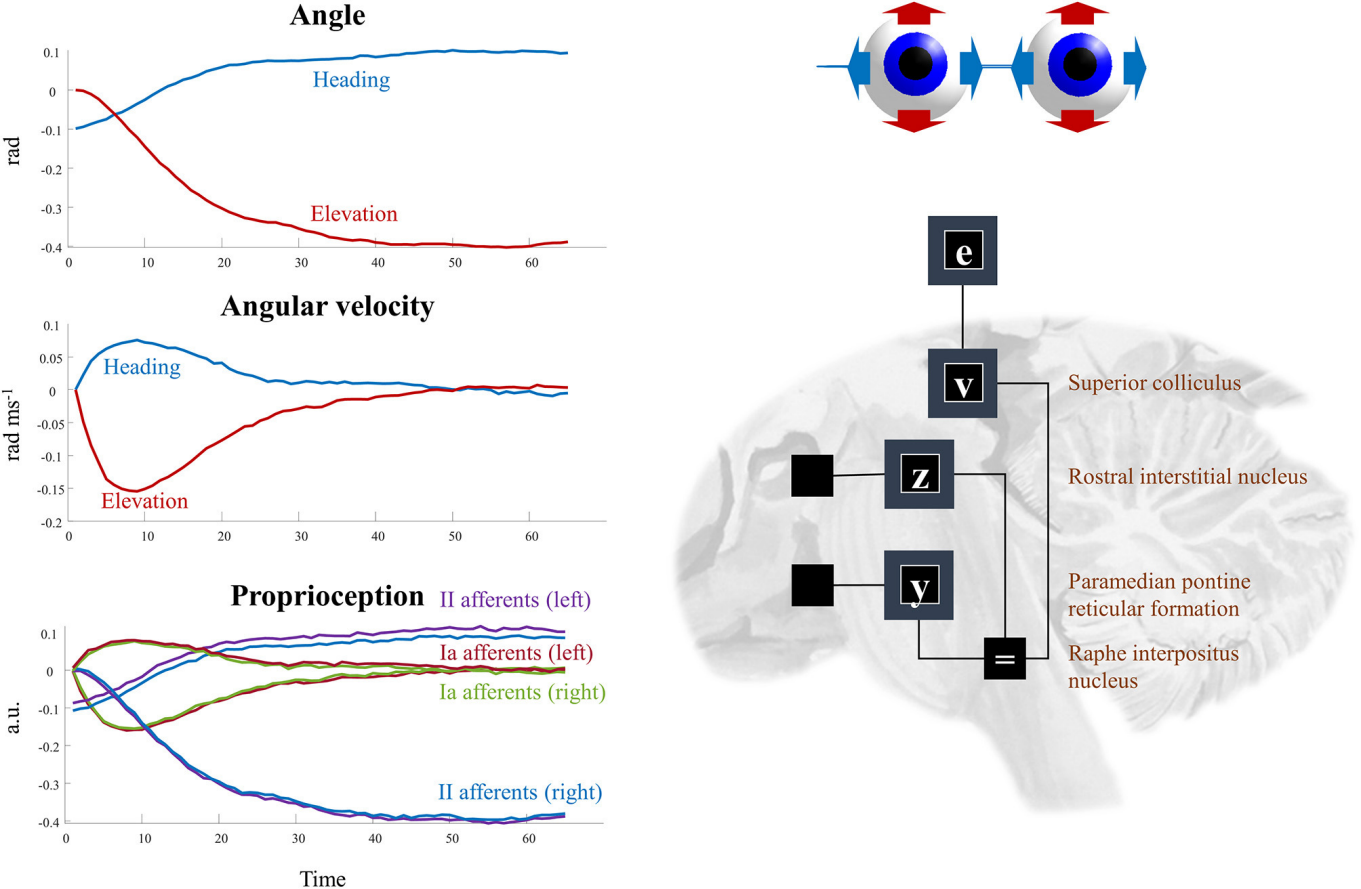
Oculomotion. The plots on the left of this figure show an example of the kinds of dynamics that result from Equations (9–12). These illustrate a single saccade toward some equilibrium point determined by factor **v**. The first two plots detail the hidden states, comprising the heading angle of the eyes, their elevation, and the rates of change of each of these. In addition, the third plot illustrates the proprioceptive data we might expect these dynamics to generate. These are divided into sensory neurons that report instantaneous muscle tendon stretch (II afferents) and those that report changes in this (Ia afferents) for the right and left eye. Note that these differ only in the heading angle—as eye movements are congruent. The constant discrepancy in the heading angle results from the angle of convergence of the eyes. On the right, the factors are arranged to be consistent with the brainstem structures that deal with saccades in the vertical (factor **z**) and horizontal (factor **y**) directions. The proprioceptive signal is expressed in arbitrary units (a.u.) which could be converted to firing rates with the appropriate (e.g., sigmoidal) transforms.

The superscripts here refer to the factors determining the prior densities of each variable in the graph of Figure 5. The precision matrices Π stand for inverse covariances. Each of these factors can itself be factorized (assuming diagonal precision matrices) into elevation and heading angles and into left and right eyes. The oculomotor brainstem is well-suited to implementing this part of the forward model (and its inversion). The superior colliculus^4^ projects to the raphe interpositus nucleus (Gandhi and Keller, 1997; Yoshida et al., 2001), and via this structure to two nuclei that represent the first (elevation and heading) factorization. The paramedian pontine reticular formation mediates horizontal saccades (Strassman et al., 1986), while the rostral interstitial nucleus of the medial longitudinal fasciculus mediates vertical saccades (Büttner-Ennever and Büttner, 1978). These nuclei then project to the cranial nerve nuclei that communicate directly with oculomotor muscles. The cranial nerve nuclei on the right of the midbrain connect to the muscles of the right eye, and those on the left connect to the left eye. This represents the second factorization into left and right eyes. Figure 6 shows how this factorization may manifest anatomically and illustrates the proprioceptive data we would anticipate on simulating the dynamics outlined above.

This just leaves the question as to where the equilibrium point (*x*^*v*^) comes from. As we have said, the superior colliculus—a midbrain structure—is an important junction in the descending pathway to the oculomotor brainstem. Via factor **v**, the dynamics depend upon factor **e**, which is the same variable that appears in our frontal eye fields in Figure 3. The frontal eye fields project to the superior colliculus (Künzle and Akert, 1977; Hanes and Wurtz, 2001), as shown in Figure 6. However, factor **e** is conditioned upon the policy, implying we may have several alternative equilibrium points available to the superior colliculus. To adjudicate between these, we need another input to the colliculus that selects between policies. We have previously argued that the output nuclei of the basal ganglia could fulfill this role (Parr and Friston, 2018c). This is consistent with the projections from the substantia nigra pars reticulata to the superior colliculus (Hikosaka and Wurtz, 1983). The selection between alternative policies is the focus of section The basal ganglia. A similar analysis could be made of head movements and the vestibular data they generate. We omit this here to avoid duplication of the concepts outlined above. More generally, selecting a series of attracting points, as we have for saccadic eye movements, offers a useful way of representing environmental dynamics, including those that are out of our control. For instance, by replacing the static prior over object location with a series of transition probabilities, we could predict the next location given the current location. This converts the static elements of the model into a hidden Markov model. By associating each possible location with an attracting point, we can predict the continuous trajectories of the object as it is drawn from one location to the next (Huerta and Rabinovich, 2004; Friston et al., 2011). This style of dynamical modeling for active inference has been exploited in the context of a 2-dimensional visual search task (Friston et al., 2017a), and in control of arm movements in 3-dimensions (Parr et al., 2021).

### The Basal Ganglia

In thinking about the problem of where to look, we must consider a set of subcortical nuclei known to play an important role in planning (Jahanshahi et al., 2015). The basal ganglia receive input from much of the cerebral cortex and provide output to the superior colliculus, among other structures. This means they are well-positioned to evaluate alternative action plans based upon the beliefs represented by the cortex, and to modulate the cortical projections to the colliculus to bring about the most likely eye movements. As such, these nuclei have frequently been associated with inferences about what to do in the process theories associated with active inference (Friston et al., 2017a,b; Parr and Friston, 2018b).

What makes one eye-movement better than another? One way to think about this is to frame the problem as one of experimental design (Itti and Koch, 2000; Friston et al., 2012). The best experiments (or eye movements) are those that maximize expected information gain^5^—i.e., the mutual information (Lindley, 1956) between data (*y*) and hypotheses or causes (*x*) under some design or policy (π):

(13) $$\begin{array}{l} \begin{array}{l} 𝕀\left[X,Y|\pi \right]=D_{KL}\left[P\left(x,y|\pi \right)||P\left(x|\pi \right)P\left(y|\pi \right)\right]\\ ={𝔼}_{P\left(y|\pi \right)}\underset{\text{Information gain}}{\underset{︸}{\left[D_{KL}\left[P\left(x|y,\pi \right)||P\left(x|\pi \right)\right]\right]}}\\ =\underset{\text{Predictive}\;\text{Entropy}}{\underset{︸}{H\left[P\left(y|\pi \right)\right]}}-\underset{\text{Expected Ambiguity}}{\underset{︸}{{𝔼}_{P\left(x|\pi \right)}\left[H\left[P\left(y|x,\pi \right)\right]\right]}} \end{array} \end{array}$$

Equation (13) shows three different expressions of the mutual information, incorporating KL-Divergences—quantifying how different two distributions are from one another—and entropies. An entropy (*H*) is a measure of the dispersion or uncertainty associated with a probability distribution. The first line says that the expected information gain is greatest when the joint distribution of data and their causes, under a given policy, is very different from the product of the two marginal distributions. The second line expresses this in terms of the expected update from prior to posterior—i.e., the information gain. The third line breaks this down into two components. These are easiest to understand when thinking about what makes a good experiment. The first thing is that it should tell us something we do not already know. An experiment for which we can already confidently predict our measurements is a poor experiment. Such experiments are penalized by the predictive entropy term, which favors those experiments for which the predicted measurements are maximally uncertain, i.e., not known beforehand.

**Figure 7:**
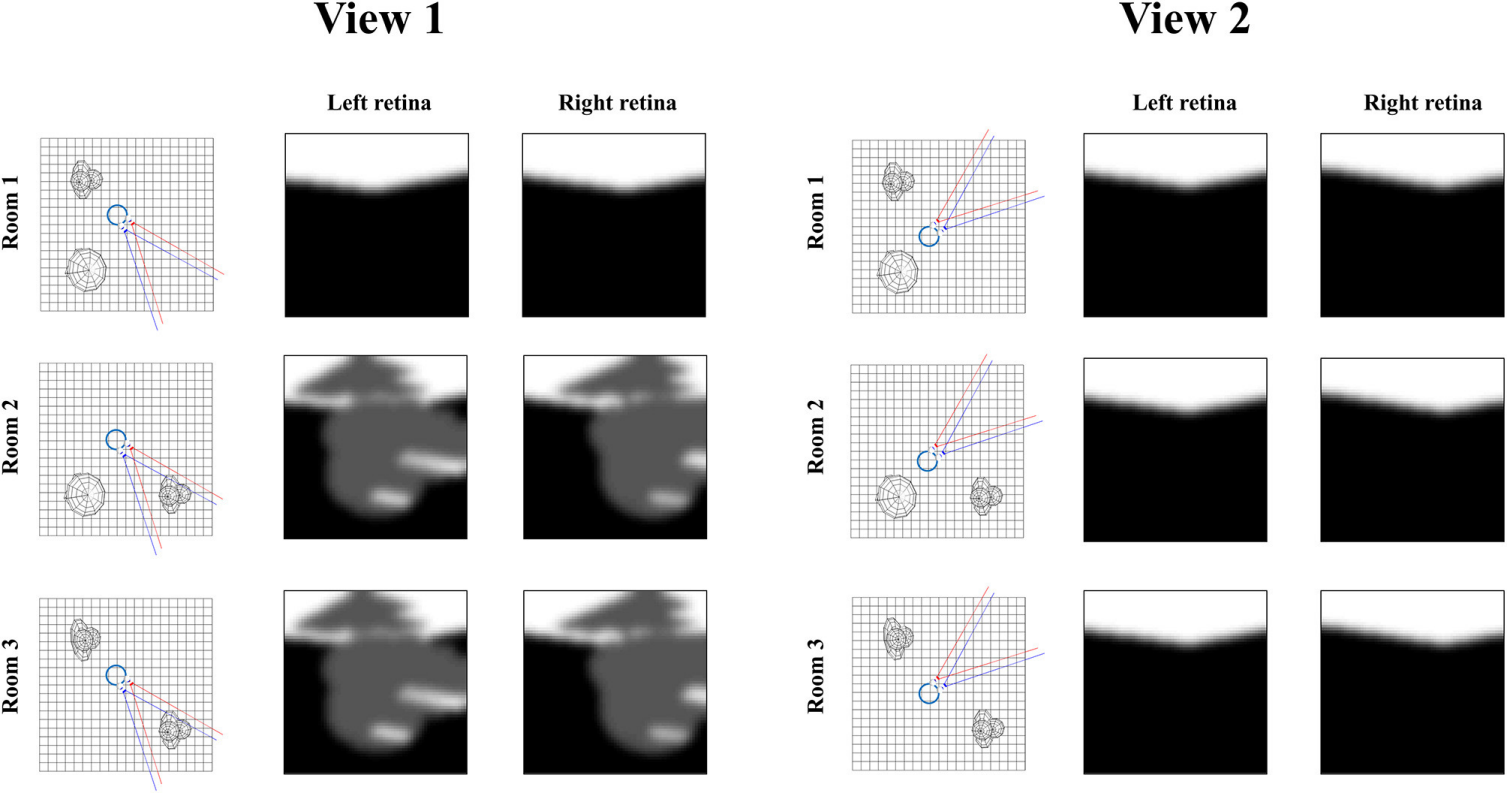
Expected information gain. This figure highlights the role of predictive entropies in adjudicating between salient actions. This shows two alternative fields of view we could choose between, through making eye or head movements. If we were not sure which room we were in, view 1 (toward the southeast corner) would be associated with a high predictive entropy, and is consequently useful in resolving uncertainty. In contrast, view 2 (toward the northeast corner) has zero predictive entropy, and does not help distinguish between rooms.

Figure 7 illustrates the relevance of the predictive entropy in adjudicating between alternative fields of view. This shows two (of many) possible head-directions and the visual input this generates in each of the three rooms shown in Figure 2. Imagine we are uncertain about the room we occupy, but relatively confident about everything else. View 1 could give rise to a view with no object, or with object 1. We can be confident that view 2 will always lead to a view with no object, as none of the three rooms have an object in this location. Any actions leading to view 1 (by moving eyes or head) will be associated with a higher predictive entropy than actions leading to view 2 (zero entropy). Intuitively this is sensible, as we will be able to tell from the consequences of view 1 whether we are in room 1, or in room 2 or 3. We will gain no information about the room from view 2. Once we have seen object 1 in view 1, we know we are in room 2 or 3, and there is no added information available in this view. We would always anticipate seeing the same thing here. At this point, the southwest or northwest corners of the room may become more salient, allowing disambiguation between the rooms that are still plausible.

The expected ambiguity term in Equation (13) expresses the fact that, even if sensory input is unpredictable, it is not necessarily useful. Everything else being equal, expected ambiguity underwrites the imperative to sample precise and unambiguous visual sensations. Perhaps the simplest example is keeping our eyes open. When our eyes are closed (or the lights are off), the probability of every retinal cell firing is roughly the same, which corresponds to a maximally ambiguous state of affairs.

The basal ganglia appear to be key in quantifying information gain (Sheth et al., 2011; White et al., 2019). However, they are part of a broader network of regions involved in making these decisions. This is important, in the sense that information gain is a functional (function of a function) of beliefs. As such, the broad range of inputs to the basal ganglia from the cortex and elsewhere may give them access to these beliefs across different modalities. This is evidenced by disorders of salience attribution, like sensory neglect syndromes (Husain et al., 2001; Fruhmann Berger et al., 2008; Parr and Friston, 2017a)—which occur with lesions to the superior longitudinal fasciculus (c.f., Figure 3) (Bartolomeo et al., 2007, 2012) in addition to basal ganglia structures (Karnath et al., 2002). In the context of active vision, at least, the basal ganglia appear to be the point at which the most epistemically valuable saccadic movements are determined, given the direct influence of this subcortical network over the superior colliculus (Hikosaka and Wurtz, 1983).

## Related Work

While we have focused upon the sort of generative model the brain could employ, we have neglected the question as to how a model of this sort might develop in the first place. Prominent approaches to learning of such models from machine vision include capsule networks (Sabour et al., 2017) and the Generative Query Network (GQN) (Eslami et al., 2018). The former is a supervised learning technique in which capsules, groups of neurons representing attributes of an entity causing visual data, optimize their connections between multiple convolutional layers to associate images with their labels. The latter is an unsupervised learning approach—reminiscent of a variational autoencoder (Kingma and Welling, 2013; An and Cho, 2015)—that learns two functions. The first is a function from observations to a representation of a scene and the second is a generative function that predicts observations, in a viewpoint-dependent manner, under the current scene representation. The two are jointly optimized based upon the fidelity with which observations are predicted given the scene representation. While unsupervised in the sense that no labeled training data are used, this approach could be viewed as supervised learning of a function from viewpoint to visual data.

There are important shared features between the generative model presented in this paper and those that emerge from training capsule networks or the GQN. Perhaps the most striking is the importance of factorization. In capsule networks, factors are an integral part of the network. Each neuron within a capsule represents distinct features in relation to other neurons. This allows a capsule—representing a given object—to represent that object in multiple orientations, or colors. In the GQN, factorization emerges from training on environments in which different attributes can vary independently. For instance, training on views of red cubes, red triangles, and blue spheres enables reconstruction of, previously unobserved, red spheres. In this paper, we have highlighted the factorization of different explanatory variables (i.e., latent causes) that manifest in different visual streams—for instance, changing our viewpoint does not change object identity, and vice versa.

A second shared feature is the increase in the spatial scale of receptive fields, as we move from observations to their causes. In capsule networks, this arises from their convolutional architecture. In our generative model, the convergence of high dimensional pixel spaces through to hidden layers with fewer and fewer units is represented, in reverse, by the generation of objects from scenes, surfaces from objects, and pixel intensities from surfaces.

Given that there are successful machine learning approaches available—that effectively learn the structure of a generative model for visual rendering—it would be reasonable to ask what is added by the approach pursued here. In short, the benefit is transparency, in the sense of both explainability and interpretability (Marcinkevičs and Vogt, 2020). The benefits of approaches based upon deep learning are that they scale well, and that the models they learn emerge from the statistical regularities in the data on which they are trained. However, the interpretability of the resulting models is not always straightforward. In contrast, specifying an explicit generative model affords an explicit interpretation of the ensuing inferences. This may not matter when developing new approaches to visual rendering but is crucial in advancing hypotheses as to how the brain (and other sentient artifacts) solves active vision problems. The account advanced in this paper is not designed to replace machine learning but offers an example of the kind of generative model they might implicitly learn.

## Discussion

In this paper, we set out a generative model capable of generating simple retinal images. Our aim was to determine the set of explanatory variables the brain could call upon to explain these visual data, the dependencies between these variables, and the anatomical connectivity that could support the requisite neuronal message passing. In other words, we sought to identify the problem the visual brain must solve. From a neurobiological perspective, one conclusion we could draw from this analysis is that few parts of the brain are not involved in active vision.

We have seen how beliefs about scenes, and the objects in those scenes, thought to be represented in the temporal lobe, are combined with beliefs about the retinal location. The latter depend upon the parietal cortices and their relationship with medial temporal and frontal lobe structures. If we know the retinal location and the set of surfaces in a scene, we can compute which surfaces lie within our field of view and determine (for a given light source) the influence of those surfaces on retinal cells. This is the retinocortical pathway in reverse. Explanations of visual data afforded by a model of this sort are highly sensitive to where the retina is. This means part of the explanation must always include our choices about where we position our retina. Central to this is the computation of expected information gain, which implicates the oculomotor loops of the basal ganglia. In addition, the process of acting to change our eye (or head) position—when viewed as an inference problem—requires that we predict all of the sensory consequences of the action we hope to execute. We detailed how this could play out in the oculomotor brainstem, predicting the proprioceptive data we hope to realize.

Clearly, there are limitations to the model presented here, and many aspects of vision that are not accounted for. It is useful to consider how these could be incorporated in this generative model. First, there are other ways, in addition to moving our eyes, in which we can influence our visual environment. For instance, we could move our hands in our field of view (Limanowski and Friston, 2020). We could go further and move objects around in the environment or assume that other agents can do so. This means unfolding the prior beliefs from Figure 1 in time, such that they factorize into a series of policy-dependent transition probabilities. Time-dependence adds an interesting twist to the expected information gain, as it means that the posterior predictive entropy grows over time for unobserved locations. The reason for this is simple. The longer the time since looking in each location, the greater the probability that something has changed. This is consistent with Jaynes' maximum entropy principle (Jaynes, 1957). The result is a form of inhibition of return (Posner et al., 1985), the duration of which varies with the precision of probabilistic transitions over time (Parr and Friston, 2017b). The duration of this inhibition of return is one of the crucial differences between static and dynamic environments: reflecting the possibility that things have changed since each location was last fixated. This engenders loss of confidence about state of affairs at that location—and an epistemic affordance of return that increases with time. This relates to other visual phenomena, even in the absence of overt eye movements. Periodic redirection of covert attention—a form of mental action (Rizzolatti et al., 1987; Hohwy, 2012; Limanowski and Friston, 2018)—based upon the accumulated uncertainty of unattended features reproduces binocular rivalry phenomena (Parr et al., 2019), in which perception alternates between different images presented to each eye (Leopold and Logothetis, 1999; Hohwy et al., 2008).

We have omitted interesting questions about texture and color vision. Textured surfaces could be modeled through varying the constants (*c*_1_, *c*_2_, *c*_3_) from Equation (6) and the ambient lighting (α) as functions of their location on a surface. Color vision could be incorporated simply by repeating section The Retinocortical Pathway for several different wavelengths of light—specifically, the red, green, and blue wavelengths detected by different cone photoreceptors (Nathans et al., 1986). This would aid in disambiguating the roles of magnocellular and parvocellular streams, involved in dissociable aspects of trichromatic and monochromatic vision (Masri et al., 2020). The magnocellular stream also seems to have a key role in detecting motion (Merigan et al., 1991) – something that is highly relevant in the context of active event recognition (Ognibene and Demiris, 2013).

From a computational perspective, there are important outstanding questions about the role of precision (i.e., neuromodulation) which may involve second order thalamic nuclei, like the pulvinar (Kanai et al., 2015), and the cholinergic basal nucleus of Meynert (Moran et al., 2013). These could be accommodated in this model through including prior beliefs about the precision or variance associated with regions of the visual field. This may be particularly relevant in understanding how subcortical structures participate in visual perception. For instance, the role of the amygdala in enhancing the perception of fearful faces (Pessoa et al., 2006; Adolphs, 2008) could be formulated as inferences about the precision of visual features consistent with this emotional state. Another important computational feature was omitted in our discussion of models of oculomotion. We neglected to mention the role of generalized coordinates of motion (acceleration, jerk and higher order temporal derivatives) (Friston et al., 2010), which offer a local approximation to the trajectory of dynamical variables, as opposed to an instantaneous value. This has important implications for things like sensorimotor delays (Perrinet et al., 2014), accounting for small discrepancies in the time the brainstem receives a proprioceptive signal compared to the time an oculomotor muscle contracted. In brief, representations of the local trajectory enable projections into the immediate past or future. To see how generalized coordinates of motion can be incorporated into a factor graph, see (Friston et al., 2017a).

Why is it useful to formulate a generative model of active vision? There are several answers to this question. The first is that having a forward model is the first step in designing an inference scheme that inverts the model. This is a matter of undoing everything that was done to generate visual data, so that their causes can be revealed. There have been promising advances in practical, scalable, model inversion for active vision from a robotics perspective, that use deep neural networks to learn a generative model that predicts camera images (Çatal et al., 2020), leading to Bayes optimal behavior in a real environment. Similar approaches have been developed both in the visual domain (Fountas et al., 2020; van der Himst and Lanillos, 2020), and in a generic (non-visual) control setting, which may also have applications for high-dimensional visual data (Tschantz et al., 2020). By treating vision as active, we can design agents that actively sample the environment to resolve their uncertainty, in high-dimensional, incongruent settings. This takes us beyond static deep learning models which, although apt at simple classification tasks (LeCun and Bengio, 1995; Jin et al., 2017), are unable to handle the complexity involved in human active vision.

The second is that this model generates behavior (i.e., saccades). As we highlighted in section The Basal Ganglia, the saccades performed depend upon prior beliefs. This means measured eye movements could be used to draw inferences about the parameters of prior beliefs in the model used by an experimental participant, or clinical patient (Mirza et al., 2018; Cullen et al., 2020). Virtual reality technologies offer a useful way to investigate this, with tight control over the visual environment combined with eye-tracking (Limanowski et al., 2017; Harris et al., 2020a,b). In principle, we could present visual data consistent with the generative model set out here and use this to test hypotheses about the structure of the generative model used by the brain, or about the parameters of each factor. One such hypothesis as to the anatomical implementation has been set out in the figures. However, it is important to recognize that this is one of many hypotheses that could have been advanced. Crucially, a generative model for visual data allows us to generate stimuli that vary according to specific hidden causes. This would allow for alternative anatomical hypotheses to be evaluated through neuroimaging, as we would anticipate variation in a given hidden state should lead to variation in beliefs about this state, and changes in neural activity—i.e., belief updating—in those regions representing these beliefs.

The third utility of forward models of this sort is that understanding the conditional dependencies in a model, and by implication the structure of the neuronal message passing that solves the model, we have an opportunity to frame questions about classical disconnection syndromes (Geschwind, 1965a,b) in functional (computational) terms (Sajid et al., 2020). We have briefly touched upon some of these syndromes, including visual field defects, agnosia, and neglect. Generative models of active vision let us express the mechanisms that underwrite these syndromes in the same formal language—that of aberrant prior beliefs. This approach is commonly used to characterize inferential pathologies in computational psychiatry (Adams et al., 2015).

## Conclusion

Under modern approaches to theoretical neurobiology—including active inference—brain function is understood in terms of the problems it solves. Its biology recapitulates the structure of this problem. In this paper, we have attempted to define the problem faced by the active visual system. This is framed as explaining visual input, where good explanations involve not just the external environment, but how we choose to position our sensors (i.e., retinas) in that environment. This explanation takes the form of a predictive model comprising factors that determine the geometry of objects expected in a given room, the placement of the retina in that room, and the combination of these variables in generating a retinal image. The factors involved in determining the placement of the retina can be further unpacked in terms of their causes—i.e., the most epistemically rich saccades—and their consequences for the dynamics of, and proprioceptive inputs from, the eyes. We hope that this paper provides a useful reference that brings together the probabilistic models required for aspects of biological active vision.

## Data Availability Statement

The datasets presented in this study can be found in online repositories. The names of the repository/repositories and accession number(s) can be found below: https://github.com/tejparr/Generative-Models-Active-Vision.

## Author Contributions

All authors listed have made a substantial, direct and intellectual contribution to the work, and approved it for publication.

## Conflict of Interest

The authors declare that the research was conducted in the absence of any commercial or financial relationships that could be construed as a potential conflict of interest.

## Footnotes

^1^If *v* contains the four vectors corresponding to the vertices of a quadrilateral surface, then *v*_*n*_ is obtained (with appropriate normalization) as *v*_*n*_∝(*v*_1_−*v*_2_) × (*v*_4_−*v*_2_).

^2^Unless you are a chameleon: Katz et al. (2015).

^3^This assumption of conjugacy may underwrite internuclear ophthalmoplegia. This is a syndrome—caused by brainstem demyelination or stroke—in which the predictions required for one eye to move towards the nose (while the other moves away from it) are interrupted. This violation of the conjugacy assumption has consequences for the contralateral eye, which exhibits a pathological oscillatory nystagmus.

^4^The superior colliculus exhibits a log-polar retinotopy which implies the *x*^*v*^ variable might be represented in this coordinate system. The functional relevance of this is that the probability density for *x*^*v*^, when translated into polar or Cartesian coordinates, will assign higher variance to more eccentric values. This has been proposed as an explanation for the increased variance of saccadic endpoints for more eccentric locations in a Cartesian frame, despite unform variance in log-polar reference frames (Daucé and Perrinet, 2020).

^5^From the perspective of active inference, this is normally augmented with an additional distribution that ascribes greater probability to preferred datapoints, turning the mutual information into an expected free energy. However, we focus upon information seeking specifically, under the assumption that eye movements are primarily exploratory (i.e., preferences over visual data are uniform). This is a special case of an expected free energy.
